# Evolution of toll-like receptors in the context of terrestrial ungulates and cetaceans diversification

**DOI:** 10.1186/s12862-017-0901-7

**Published:** 2017-02-16

**Authors:** Edson Ishengoma, Morris Agaba

**Affiliations:** 1 School of Life Science and Biongineering, The Nelson Mandela African Institution of Sciences and Technology, P.O. Box 447, Arusha, Tanzania; 2 Mkwawa University College of Education, University of Dar es Salaam, P.O. Box 2513, Iringa, Tanzania; 3 Biosciences Eastern and Central Africa, International Livestock Research Institute, Nairobi, GPO00100 Kenya

**Keywords:** Toll-like receptors, Giraffe, Terrestrial ungulates, Cetaceans, Adaptive evolution, Functional variation

## Abstract

**Background:**

Toll-like receptors (TLRs) are the frontline actors in the innate immune response to various pathogens and are expected to be targets of natural selection in species adapted to habitats with contrasting pathogen burdens. The recent publication of genome sequences of giraffe and okapi together afforded the opportunity to examine the evolution of selected TLRs in broad range of terrestrial ungulates and cetaceans during their complex habitat diversification. Through direct sequence comparisons and standard evolutionary approaches, the extent of nucleotide and protein sequence diversity in seven Toll-like receptors (TLR2, TLR3, TLR4, TLR5, TLR7, TLR9 and TLR10) between giraffe and closely related species was determined. In addition, comparison of the patterning of key TLR motifs and domains between giraffe and related species was performed. The quantification of selection pressure and divergence on TLRs among terrestrial ungulates and cetaceans was also performed.

**Results:**

Sequence analysis shows that giraffe has 94–99% nucleotide identity with okapi and cattle for all TLRs analyzed. Variations in the number of Leucine-rich repeats were observed in some of TLRs between giraffe, okapi and cattle. Patterning of key TLR domains did not reveal any significant differences in the domain architecture among giraffe, okapi and cattle. Molecular evolutionary analysis for selection pressure identifies positive selection on key sites for all TLRs examined suggesting that pervasive evolutionary pressure has taken place during the evolution of terrestrial ungulates and cetaceans. Analysis of positively selected sites showed some site to be part of Leucine-rich motifs suggesting functional relevance in species-specific recognition of pathogen associated molecular patterns. Notably, clade analysis reveals significant selection divergence between terrestrial ungulates and cetaceans in viral sensing TLR3. Mapping of giraffe TLR3 key substitutions to the structure of the receptor indicates that at least one of giraffe altered sites coincides with TLR3 residue known to play a critical role in receptor signaling activity.

**Conclusion:**

There is overall structural conservation in TLRs among giraffe, okapi and cattle indicating that the mechanism for innate immune response utilizing TLR pathways may not have changed very much during the evolution of these species. However, a broader phylogenetic analysis revealed signatures of adaptive evolution among terrestrial ungulates and cetaceans, including the observed selection divergence in TLR3. This suggests that long term ecological dynamics has led to species-specific innovation and functional variation in the mechanisms mediating innate immunity in terrestrial ungulates and cetaceans.

**Electronic supplementary material:**

The online version of this article (doi:10.1186/s12862-017-0901-7) contains supplementary material, which is available to authorized users.

## Background

Mammalian Toll-like receptors (TLRs) are membrane-bound proteins expressed in defense cells where they have evolved to mediate innate immune system through recognition of various pathogen-associated molecular patterns (PAMPs) [1, 2]. Functional classification of TLRs depends on the cellular location and the ligands they bind. For example, TLR2 is located on the outer membrane and forms dimer complex with TLR1 or TLR6 to recognize peptidoglycans, lipoproteins or lipoteichoic acid of gram positive bacteria [3, 4]. Other outer membrane TLRs include TLR4 which dimerize to recognize lipopolysaccharides (LPS) of gram negative bacteria [5], TLR5 which recognizes flagellins [6] and TLR10 which has recently been shown to have anti-inflammatory effects, and perhaps in combination with TLR2 may be associated with mycobacterial infections [7, 8]. Endosome confined TLRs includes TLR3, TLR7, TLR8 and TLR9. TLR3 recognizes double-stranded RNA (dsRNA), TLR7 and TLR8 are activated upon contact with single-stranded RNA (ssRNA) while TLR9 recognizes CpG DNA from virus, fungi and other invading pathogens [9–11].

TLRs share the same basic architecture comprising of a large extracellular domain (ECD) responsible for PAMP binding, a single-pass trans-membrane (TM) domain believed to play a role in membrane receptor stabilization and receptor-receptor oligomerization [12] and an intracellular Toll/interleukin-1 receptor (TIR) domain responsible for intracellular signal transduction and orchestration of cellular responses [1, 13]. The extracellular portion contains Leucine-rich repeat (LRR) motifs, which are arrays of 20–30 amino acid-long protein sequences enriched with the hydrophobic amino acid Leucine. Activation of TLRs by ligands initiates a cascade of events that leads the TIR domain to engage TIR domain-containing adaptor proteins. Adaptors proteins such as myeloid differentiation primary-response protein 88 (MYD88) or TIR domain-containing adaptor protein inducing IFNβ (TRIF) play a role in linking TLRs to nuclear transcription factors [2]. Such transcription factors include activator protein 1 (AP-1), Interferon regulatory factors (IRFs) or Nuclear Factor kappaB (NF-kB) which induce production of pro-inflammatory factors to mediate immune responses.

The evolution of TLRs is believed to have ancient and complex history that can be traced back to basal metazoans like sponges, Hydra (*Hydra magnipapillata*) and sea anemone (*Nematostella vectensis*) [14, 15]. Episodes of gene duplication, gene loss and gene conversion appear to have produced different TLR repertoire and functional diversification in various vertebrate species [16, 17]. The vertebrate ancestor at least possessed fifteen TLR members: TLR1-5, 7–9, 11–14, 19, 21, and 22 [18, 19]. Comprehensive phylogenetic studies of vertebrate TLRs reveal complete absence of TLR21 and TL22 and rarity of TLR12-14 and TLR 19 in land-dwelling vertebrates suggesting that pervasive TLR gene loss has taken place during transition from water to land [18, 20]. Even among mammals, TLRs are present in varying numbers; for example, primates and ungulates have ten TLRs [21–23] while some rodents (e.g. *Mus musculus*) possess 12 TLRs [14]. Novel TLRs combination in different animal groups reflects the need to acquire elaborate and efficient system to recognize and respond to diverse pathogens presented by different environments.

Conventionally, genes involved in immunity should exhibit an accelerated evolutionary rate indicative of adaptive struggle between host and the invading pathogens. However, various studies have found TLRs to be evolutionarily conserved within and between lineages [24–26]. Nevertheless, characterizing TLRs according to their domains provides finer resolution on the role of natural selection on TLRs. Several studies have shown purifying selection dominating in TLR regions responsible for oligomerization while considerable degree of variation was observed in TLR regions responsible for PAMP binding [27]. Other studies taking individual species, specific taxa and location of receptor into account have found evidence of adaptive substitutions in bovine TLR2 and TLR5 [28, 29] and TLR4 in primates [30, 31].

In a recently published work in our group, we identified genes associated with innate immunity to have been overrepresented among positively selected genes in the giraffe lineage when compared to okapi and cattle [32]. Giraffes are generally susceptible to viral and bacterial infections such as rinderpest [33], anthrax [34] and tuberculosis [35], that also affect all wild and livestock ruminants. Repeated exposure to infectious agents may result in extinction or adaptation of species [36], signs of the latter expected to be detected on genes especially those mediating defense against infections. Furthermore, members of the family Giraffidae have diverged over extended evolutionary periods in contrasting environments: giraffes occupy the trypanosome infested savannah while okapi is restricted to serene Congo forests suggesting that differential adaptation in response to infectious agents may be expected between the two species.

In a broader context, giraffe and okapi are members of a diverse group of terrestrial ungulates with wide zoogeographic distribution and exhibiting remarkable diversity in size, diet and habitat. The diversity of ecological specializations in ungulates seems to be attributed to habitat changes during the so-called Eocene Climatic Optimum approximately 40 million years ago. The period was also accompanied by the emergence of different habitat niches creating possibilities for variety of body forms and dietary innovations [37]. This diversification phenomenon was even more pronounced in ruminants which have displayed an extraordinary variety of body sizes and diets ranging from very small (<20 kg) and diet generalists (e.g. dik dik) to very large (>700 kg) and diet specialists (e. g. giraffe). Occupation of varied ecological niches and dynamic dietary preferences presented challenges in finding symbiotic balance between the host immune system and endemic rumen microbial population [38, 39]. Long term, this immune system–microbiota relationship may allow for the species- and/or niche-specific adaptations in the development and maintenance of regulatory homeostasis in response to pathogenic invasion.

Closely related to terrestrial ungulates are cetaceans, including whales, dolphins, and porpoises, which, by contrast, are a group of secondarily adapted marine mammals with a history of terrestrial occupation before re-colonizing aquatic habitats [20, 40]. In addition to anatomical and physiological innovations required for life in water [41], cetaceans must have been confronted with even more formidable challenges from ever-changing water-borne pathogens.

We hypothesized that adaptive evolutionary pressure mediated by infectious agents due to ecological diversity has contributed to the evolution of TLR diversity in species with complex evolutionary history as exemplified by ungulates and cetaceans. To understand the extent of functional variation in the genes modulating innate immunity in this group, we have taken advantage of the availability of giraffe and okapi genomes to identify seven TLRs and examine adaptive sequence changes in comparison with other related species. The ultimate goal is to gain insight on the adaptive pressure on the innate immune system associated with the divergence of terrestrial ungulates and cetaceans.

## Methods

### Species and sequences

Giraffe and okapi TLR sequences (Additional file 1) were sequenced as part of the giraffe genome project [32]. The TLR sequences of cetaceans and other artiodactyls used in the analyses were retrieved from Reference Sequence (RefSeq) database of the National Center for Biotechnology Information (NCBI) (www.ncbi.nlm.nih.gov) or Ensembl at the European Bioinformatics Institute (www.ensembl.org). For sequences obtained from NCBI, identification of putative TLR orthologs for the target species was achieved using BLAST against RNA RefSeq database. BioMart was used to extract orthologs for sequences obtained from Ensembl. For each TLR included in the study, giraffe, okapi and a subset of other species in *Cetartiodactyla* including at least one species from an outgroup taxon (horse, rhino or both), were used in the analysis. To qualify for inclusion in the analysis the sequences had to have complete coding length in all species considered. Thus, TLR1, TLR6 and TLR8 were not considered for analysis as they were either not successfully sequenced or were of partial sequences in giraffe and okapi. Moreover, to ensure reliability of protein coding quality for each of the TLR in the target species, their sequences should have had no any internal termination codon. For example, baiji dolphin (*Lipotes vexillifer*) TLR5 sequence was found to have internal stop codon and was removed from subsequent analysis as it was not known whether in this species the sequence is a pseudogene or a result of a sequencing error. The final list of species used for each TLR and their Ensembl identity or accession numbers, excluding giraffe and okapi, are presented in Additional file 2. Protein translation of TLRs coding sequences were aligned using MUSCLE [42], back-translated using RevTrans [43] and phylogenetic trees constructed using PhyML [44].

### TLRs sequence and motif comparison

A web based simple modular architecture research tool (SMART) utilizes Hidden Markov models to query a collection of well annotated domain families associated with wide variety of nuclear, signaling and extracellular proteins [45]. The structural organization of TLR domains in the studied TLRs was analyzed using SMART. Web based LRRfinder is derived from a large database of unique, naturally occurring LRRs (tLRRdb) allowing the identification of not only highly conserved LRR sequences, but also those which uniquely deviate from the commonly described LxxLxLxxN/CxL consensus [46]. In this study, the LRRfinder was used to detect the number of LRRs present in the deduced amino acid sequences of giraffe, okapi and cattle TLRs. To identify whether there was significant difference in the number of giraffe LRRs and closely related ruminants, comparison was performed with the corresponding numbers of LRRs in okapi and cattle (Additional file 3: Table S1). In addition, comparison was performed on total number of nucleotide and amino acid sequence differences of each TLR gene among giraffe, okapi and cattle (Additional file 3: Table S1).

### Site-based analyses of positive selection

Multiple alignments of TLR sequences and corresponding phylogenetic trees were used as inputs for codon-based analysis of positive selection. We applied site-based analyses which assume that all branches in a phylogeny are evolving at the same rate but certain sites may be under differing selection pressure i.e. the individual sites may be under purifying, neutral or positive selection [47]. The analyses were implemented using CODEML program of the Phylogenetic Analysis by Maximum Likelihood (PAML) package. Different model-based tests of selection exist in PAML which generally produce equivalent results although some tests are observed to be more conservative than others [48, 49]. To increase the likelihood of detection of positively selection, we used the less conservative M7/M8 test to examine the extent of selection acting on TLRs. M7 serves as null selection model by only allowing codons to evolve neutrally or under purifying selection following a beta distribution while the alternative M8 adds an extra class of sites under positive selection. The likelihood ratio test (LRT) was applied to determine significant cases of positive selection. Significant amino acids sites under positive selection were determined using Bayes Empirical Bayes (BEB) approach with posterior probability at 95% cut-off.

Simultaneously, we applied an alternative approach based on maximum likelihood to examine the extent of evolutionary pressure occurring at every codon in all TLRs using the “site-wise likelihood ratio” method as implemented in the SLR package [50]. The SLR test consists of performing a likelihood-ratio test on site-wise basis, testing the null model (neutrality, ω = 1) against an alternative model (ω ≠ 1). The method test whether a given site has undergone selection or not, and the test statistic summarizes the strength of the evidence for selection rather than the strength of the selection itself. The sites that were predicted to undergo positive selection using M8 model were cross-checked against the sites that were predicted as significant by the SLR method. Positively selected sites that were concordantly identified by the two methods as significant were assumed to be adaptively important. These sites were mapped to human TLRs Swiss-Prot entries to determine their functional relevance based on whether they map onto key TLR domains and motifs (Additional file 4: Table S2).

### Clade models analyses of selection divergence

To identify whether divergent selection would be detected between terrestrial ungulates and cetaceans clades in their combined phylogeny, we applied PAML’s clade models. Clade Model C (CmC) partitions different branches within the phylogeny as “background” and “foreground” as well as existence of three site categories, two of which experience uniform selection across the entire phylogeny (either purifying selection (0 < ω0 < 1) or neutral evolution (ω1 = 1)) while the third is allowed to vary between background (ω2 > 0) and foreground (ω3 > 0) branches [51]. We used the recently developed null model of the CmC (M2a_rel) which does not allow the third site class to vary between two or more branch types, to test for the existence of divergent selection between terrestrial ungulates and cetacean clades [52]. In the case where significance was detected between CmC and M2a_rel, we proceeded to test for existence of positive selection between the two clades using the branch-site models [53] assuming, among other things, that the divergent site class has evolved by positive selection in the cetacean branch (ω3 > 1) while the background branches has been under the influence of purifying selection or neutral evolution.

### Structural analysis

For the TLR which showed significant selection divergence between terrestrial ungulates and cetacean clades, we were interested to determine the functional significance of specific changes in the TLR during giraffe evolution. To this end, we obtained and reviewed the crystal structure of the TLR to identify which residues are critical in the ligand-receptor interaction. Moreover, we reviewed site-directed mutagenesis studies to identify sites predicted to have any TLR functional impacts. We also performed a PolyPhen screen [54] to identify sites that are predicted to be probably functionally consequential if a substitution has taken place in a giraffe TLR when compared to closely related species. Finally, we identified positively selected sites based on BEB prediction on this TLR. Following the identification of all the important sites, we referenced giraffe TLR substitutions against the identified important sites of the TLR structure for correspondence.

## Results

### TLRs sequence and motif analysis

**Fig. 1:**
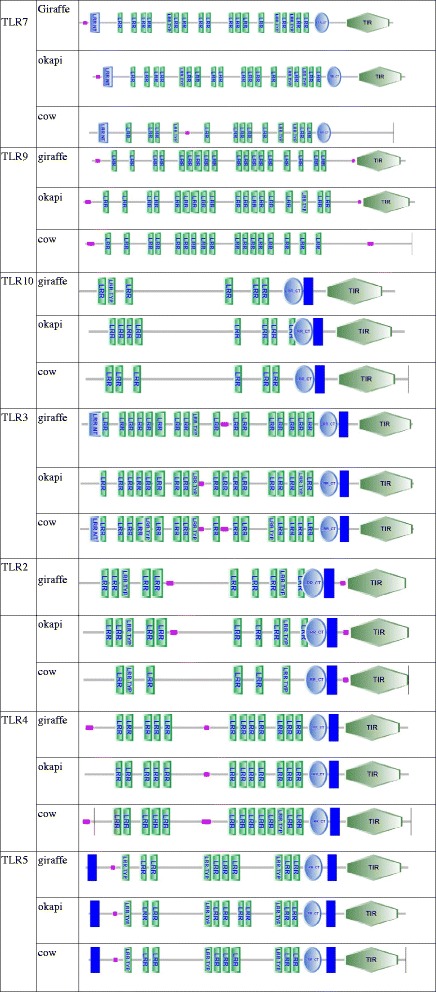
Comparison of domain architecture of TLRs in giraffe, okapi and cattle revealed no observable differences in spatial organization of major TLR domain areas (low complexity region (*pink*), LRRs and TIR)

We successfully retrieved complete coding sequence of seven TLRs (TLR2-5, TLR7 and TLR9-10) from giraffe and okapi genome sequences. The percent nucleotide and amino acid difference of the giraffe TLR coding sequences when compared with TLRs from okapi and cattle is shown in Additional file 3: Table S1. As expected, there was a small degree of nucleotide difference with okapi sequences (<2%) and 3–5% with cattle sequences, and when comparison takes into account amino acids differences, similar pattern is observed. The receptor with the highest degree of similarity among the three species was TLR7. According to SMART predictions, comparing the patterning of the ECD, TM and TIR domains of giraffe, okapi and cattle TLRs revealed no observable differences (Fig. 1). However, for some TLRs, there were variations in the predicted numbers of LRRs among the three species despite their highly conserved sequences (Additional file 4: Table S2). Giraffe is observed to have lower number of LRRs in TLR3 (21) compared to the usual number of TLR3 LRRs in mammals (23). Okapi is observed to have lower number of LRRs (19) in TLR5 compared to 21 observed in giraffe and cattle.

### Identification and distribution of selection pressure in the TLRs

**Fig. 2:**
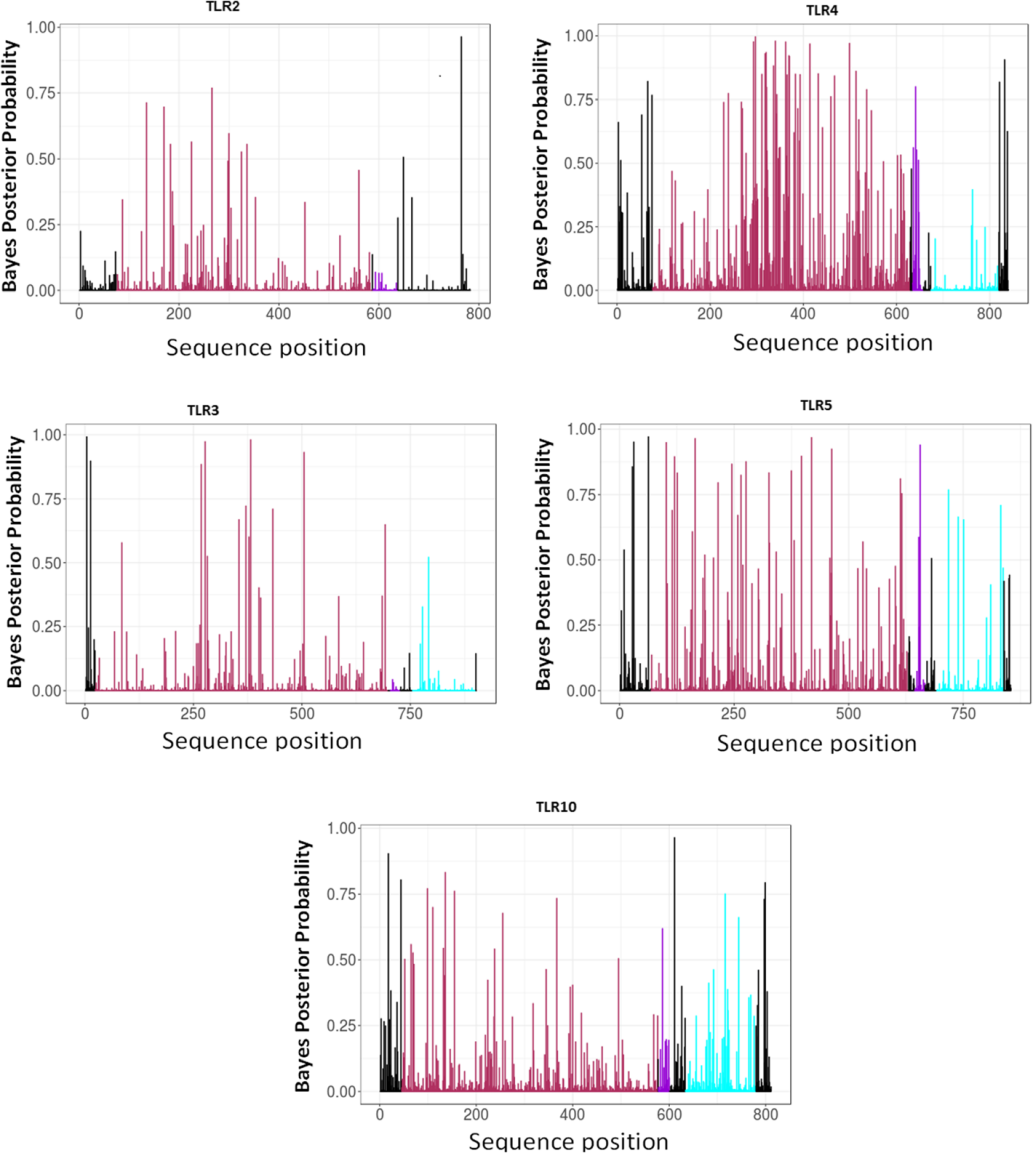
Graphical representation of distribution of selection pressure in Certatiodactyl Toll-like receptors. The majority of sites are under purifying selection. However positive selection is likely to occur in the ecto-domain (ECD) (brown-highlighted) than the transmembrane (TM) and Toll/Interleukin receptor (TIR) domain. Only TLR2-5 and TLR10 are presented for which clear positional demarcation of all three TLR domains was confidently predicted by SMART using cattle TLR as reference

The two Maximum Likelihood approaches detected evidence of positive selection in all of the TLRs studied. The results produced by M8 model indicated that the ω of all the TLR genes examined varied among codons with multiple significant codons under positive selection in five of the TLRs (Table 1). For all receptors, it was found that the proportion of sites with evidence of purifying selection (f_0_) is consistently larger than the proportion of sites with evidence of positive selection (f_1_). Thus, the majority of sites within the proteins were functionally constrained (Fig. 2). The number of positively selected codons observed for each TLR studied ranged between 23 and 113 which corresponded to 2.5–11% of the aligned codons. When significant positively selected sites based on BEB (*P* ≥ 95%) in specific TLRs are considered, TLR4 was the receptor with the highest proportion of codons under significant positive selection (13 sites), followed by TLR7 (11 sites) (Table 1). The TLR with the fewest number of positively selected sites appeared to be TLR2 and TLR10 with a single significant positive site in each of the TLRs.

**Table 1:** Parameter estimates for PAML models across TLR genes

Gene	Model	Parameter estimates	CODEML/SLR significant sites, giraffe residue, Bayes Empirical Bayes score
TLR5	M7 (β) M8 (β & ω)	*p* = 0.17, *q* = 0.26 *p* = 0.24, *q* = 0.45, f_0_ = 0.94 ω = 2.68, f_1_ = 0.06	31, L,96.1; 63,T, 97.4; 102,G, 96.3; 165,R, 97.1; 419,P, 97.36; 56, T,95.7
LR4	M7 (β) M8 (β & ω)	*p* = 0.05, *q* = 0.04 *p* = 0.23, *q* = 0.38, f_0_ = 0.90 ω = 2.59, f_1_ = 0.10	293,D,99.3; 297,A, 99.9; 318,S, 97.9; 320,E, 98.0; 336,V, 96.1; 340,V, 99.4; 362,V, 99.3; 369,F, 97.6; 370,V, 97.5; 414,V, 99.1; 499,V, 99.2; 513,T, 95.2; 832,N, 97.1;
TLR2	M7 (β) M8 (β & ω)	*p* = 0.14, *q* = 0.24 *p* = 0.22, *q* = 0.43, f_0_ = 0.96 ω = 2.55, f_1_ = 0.04	765, M, 98.4
TLR3	M7 (β)M8 (β & ω)	*p* = 0.19, *q* = 0.35 *p* = 0.28, *q* = 0.75, f_0_ = 0.98ω = 2.71, f_1_ = 0.02	4, H, 98.3; 277, V, 96.9; 382, F, 97.3
TLR7	M7 (β) M8 (β & ω)	*p* = 0.03, *q* = 0.07 *p* = 0.15, *q* = 0.59, f_0_ = 0.95 ω = 2.23, f_1_ = 0.05	100,I,97.4; 161,L,96.6; 282,I,96.5; 393,R,97.2; 461,A,95.5; 566,H,98.5; 667,L,98.8; 693,G,97.6; 697,N,96.9; 723,H,97.5; 776,N,97.1
TLR9	M7 (β) M8 (β & ω)	*p* = 0.13, *q* = 0.53 *p* = 0.17, *q* = 1.44, f_0_ = 0.93 ω = 1.72, f_1_ = 0.07	693,R,95.5; 722,K,95.7
TLR10	M7 (β) M8 (β & ω)	*p* = 0.19, *q* = 0.24 *p* = 0.35, *q* = 0.57, f_0_ = 0.94 ω = 2.28, f_1_ = 0.06	611, G, 97.1

The majority of significantly positively selected codons predicted by M8 method were also detected by the SLR methods suggesting high concordance between the two methods. Mapping of the positive selection concordant sites to annotated TLRs identified some positively selected sites to be located within the key domains and LRR motifs suggesting potential residues of adaptive significance in various species (Additional File 3: Table S1).

### Clade-specific selection divergence

Clade model test of selection divergence revealed that the majority of TLRs did not undergo selection divergence during cetaceans’ divergence from terrestrial ungulates (Table 2). However, the null hypothesis for the clade model (M2a_rel) was significantly rejected in favor of clade model C for TLR3 (LRT = 12.2, *P <* 0.001). The divergent site class in TLR3 appears to evolve under stronger positive selection in cetaceans clade with an estimated ω ≥ 4, about twice that observed in terrestrial ungulates (Table 2). In order to determine if the inference of positive selection can be made on the cetaceans’ clade as a result of selection divergence, the branch-site model was applied on TLR3 to test for the presence of positive selection on cetaceans’ clade against the background of terrestrial ungulates. Branch-site analysis did not find support for positive selection in any of the divergent clades.

**Table 2:** Results of Clade models testing for divergent selection among codons between Ungulate (Clade 0) and Cetacean (Clade 1)

	Clade model							Branch-site model	
	lnL			Site classes					
Gene	M2a_rel (np)	Clade model C (np)	LRT	0	1	2	*P*- value	LRT	*P*-value
TLR2	−8561.3 (28)	−8561.1 (29)	0.4	*P*_0_ = 0.66 ω_0_ = 0.08	*P*_1_ = 0.31 ω_1_ = 1	*P*_2_ = 0.03 ω_*Clade 0*_ = 4.6 ω_*Clade 1*_ *=*2.78	0.5		
TLR3	−9856.6 (42)	−9850.5 (43)	12.2	*P*_0_ = 0.77 ω_0_ = 0.1	*P*_1_= 0.21 ω_1_ = 1	*P*_2_ = 0.02 ω_*Clade 0*_ = 2.7 ω_*Clade 1*_ *=* 4.7	<0.001	0.000	>0.05
TLR4	−10128.7 (34)	−10128.7 (35)	0.0	*P*_0_ = 0.6 ω_0_ = 0.08	*P*_*1*_ = 0.3 ω_1_ = 1	*P*_2_ = 0.1 ω_*Clade 0*_ = 2.85 ω_*Clade 1*_ *=*2.67	0.8		
TLR5	−10467.5 (38)	−10467.3 (39)	0.3	*P*_0_ = 0.6 ω_0_ = 0.09	*P*_*1*_ = 0.3 ω_1_ = 1	*P*_2_ = 0.1 ω_*Clade 0*_ = 2.98 ω_*Clade 1*_ *=*3.71	0.6		
TLR7	−9947.7 (43)	−9946.8 (42)	1.8	*P*_0_ = 0.0 ω_0_ = 0.0	*P*_*1*_ = 0.2 ω_1_ = 1	*P*_2_ = 0.8 ω_*Clade 0*_ = 0.05 ω_*Clade 1*_ *=* 0.00	0.2		
TLR9	−10345.2 (30)	−10345.1 (31)	0.2	*P*_0_ = 0.57 ω_0_ = 0.00	*P*_*1*_ = 0.12 ω_1_ = 1	*P*_2_ = 0.31 ω_*Clade 0*_ = 0.17 ω_*Clade 1*_ *=* 0. 20	0.7		
TLR10	−8606.2 (40)	−8605.4 (41)	1.5	*P*_0_ = 0.61 ω_0_ = 0.10	*P*_*1*_ = 0.38 ω_1_ = 1	*P*_2_ = 0.01 ω_*Clade 0*_ = 4.99 ω_*Clade 1*_ *=*0.00	0.2		

The TLR with significant divergent selection was further subjected to branch-site analysis to determine if divergent selection corresponds to positive selection in the foreground (Cetacean) branch

### Mapping of important substitutions on the TLR3 structure

**Fig. 3:**
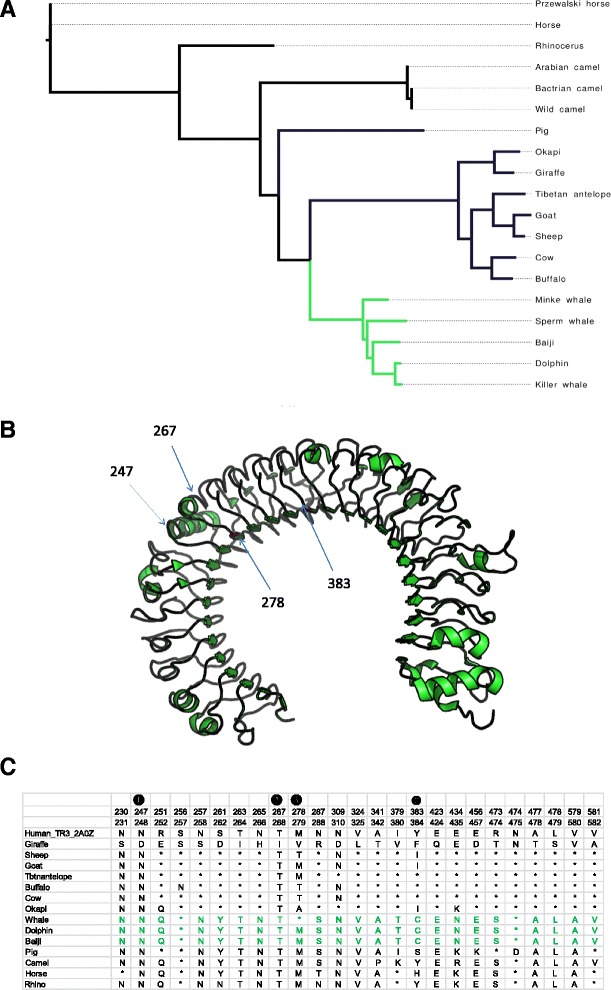
Functional prediction of important substitutions identified from giraffe TLR3. **a** A TLR3 cladogram (maximum likelihood) demarcating terrestrial ungulates (black) and cetacean (green) branches used in the clade analysis of functional divergence. **b** Crystal structure of human TLR3 ECD (PDB ID: 2A0Z) showing sites corresponding to giraffe key substitutions based on whether they are predicted to change function (PolyPhen), positive selection sites or map to empirically important site on the structure. **c** Partial alignment to show residues in other species at sites corresponding to giraffe important sites (❶ denotes TLR3 N-glycosylated site, ❷ show PolyPhen hit site and ❸ show positive selected sites based on PAML and SLR). Top number lane represents the residue position for human TLR and the second number lane is the residue position in the alignment. The asterisk (*) refers to residue identical to that of giraffe

We were still interested to find if giraffe possesses key substitutions within its TLR3 that localize to important sites of the receptor based on the crystal structure of the TLR3 ECD and site-directed mutagenesis experiments [55, 56]. First, we ensured that the observed sequence changes were not a result of sequencing errors by cross-checking if the sequences involved are identical in the two giraffes that were sequenced in the Giraffe Genome Project. Mapping of giraffe residues corresponding to sites of positive selection on the TLR3 ECD structure showed that two of these sites, Valine at position 278 and Phenylalanine at position 383 (Fig. 3b) are located on the concave side of the ECD. This concave surface was precluded by Choe et al. [55] as potential location for dsRNA ligand binding due to the presence of high amount of carbohydrates. Secondly, a PolyPhen screen on the TLR3 protein reported one unique giraffe substitution, T267I, as probably significant with a PolyPhen score of > 0.99 (Fig. 3b). However, the site does not correspond with any of the residues found in various experiments to be essential in dsRNA ligand binding. Finally, we examined the TLR3-ECD 11 N-glycosylation sites that are visible in the structure [55]. Interestingly, giraffe appears to have lost *N*-glycosylated site at position 247 where they possess Aspartate (D247) in place of conserved Asparagine (N247). A N247D mutation was shown to result in altered receptor activity in a site-directed mutagenesis experiment [56]. Therefore, similar alterations in receptor signaling may be dictated by the singular N247D change or in combination with other sequence changes in giraffe TLR3, with respect to selection divergence of TLR3 between terrestrial ungulates and cetaceans.

## Discussion

### TLR sequence analysis reveals strong conservation between giraffe and related species

This is the first study presenting the sequence analysis of 7 TLR proteins from giraffe and okapi. The protein domain prediction of the TLR sequences revealed typical TLR structure with ECD, TM and TIR domains which are similar among giraffe, okapi and cattle. The results are in accordance with previous studies on TLR gene sequences from goat and buffalo which showed a high degree of sequence similarity across species [23, 57]. The high nucleotide and amino acid similarities of giraffe TLR sequences in comparison to okapi and cattle is indicative of general conservation of TLR sequences among vertebrates in general [58]. Despite the high degree of conservation, amino acid differences did exist between species, with giraffe TLR3 showing up to 25 individual amino acid differences with okapi, giraffe's closest living relative. The comparison of giraffe LRR motifs with equivalent LRR motifs in okapi and cattle TLRs indicates similar amount of LRRs in TLRs 7, 9 and 10. The remaining TLRs showed differences in the numbers of LRRs between species, although the range of differences was not remarkable (the highest observed difference in LRRs between any pair of species was 2). This supports the importance of LRRs in TLR ligand recognition. Apparently purifying selection, perhaps due to the need to maintain TLR–ligand interaction/response system resulting from similar pathogenic pressure, has kept relatively constant the number of LRRs in various vertebrates [59].

### Recurrent positive selection has shaped the evolution of TLRs in ungulates and cetaceans

Various studies have comprehensively documented the importance of pathogen interaction and positive selection pressure in structuring diversity in the TLRs of mammalian species [31, 60]. The complex evolutionary history associated with divergence of cetaceans from terrestrial ungulates posed many pathogenic challenges, making members of this taxon interesting candidates of pathogen induced selection on immune genes. Results obtained in this study indicate that recurrent positive selection has shaped TLR evolution and diversity among terrestrial ungulates and cetaceans. Also, the observation that just small proportion of sites in all of the TLRs studied are affected by recurrent positive selection is consistent with the mostly accepted paradigm that purifying selection is the dominant force operating on TLRs [24]. Consistent with other studies, (e.g. [61]), our study noted the presence of more positively selected sites in bacterial-sensing TLRs than in viral-sensing counterparts. Viral PAMPs are ancient and conserved [62] while bacterial PAMPs are recognized on the cell surface and should accumulate new mutations fast at key residues to effectively evade recognition by the host [63]. Therefore viral infections are thought to exert stronger selective pressure than bacterial infections on immune genes, thus constraining the evolution of viral-sensing TLRs.

The bacterial-sensing TLR4 stood out as the gene with the strongest evidence of selection, in which more codons were found to be under recurrent positive selection at significant levels (Fig. 3). The high number of positively selected sites observed in TLR4 is also in line with previously reported results in primates and rodents [31, 61]. The malleability of TLR4 to selection pressure is often attributed to the capability of TLR4 to respond to a wide variety of ligands. The TLR4 forms a heterodimer complex with the myeloid differentiation factor 2 (MD2) to recognize a wide range of ligands ranging from Gram-negative bacteria LPS, yeast cell wall components, Trypanosoma and viruses [61, 64, 65]. The identification of numerous sites affected by positive selection in TLR4 in our study suggest that the diversity of ecological specializations among ungulates and cetaceans has combined with the TLR4 inherent factors to accelerate adaptive evolution of TLR4 in these species.

### Location of strong positive selection is biased in the ECDs of TLRs

The mapping of positively selected sites to the three major TLR domains shows that 92 to 100% sites were located in the ECD, a critical domain responsible for pathogen recognition. This is consistent with several recent studies conducted on primates, birds and rodents [30, 56, 61] that have noted concentration of positively selected sites in the ECD that harbors putative sites for ligand binding. The localization of many positive selection sites in the ECD, some of which are observed to be part of the LRR motifs, implies that corresponding amino acid substitutions may exert species-specific functional significance [27, 66].

### The role of terrestrial ungulates and cetaceans divergence in shaping TLRs evolution

Habitat shifts often promote adaptation and aquatic life can be considerably challenging for mammals that were originally adapted for life on land [39]. We examined patterns of TLRs in the context of terrestrial ungulates and cetaceans divergence hypothesizing that terrestrial and aquatic habitats provide contrasting environments that harbors distinct pathogenic communities. In turn, this would provide clues on specific pathogens accelerating adaptive differentiation in the immune genes operations between terrestrial-adapted ungulates and aquatic-adapted cetaceans. The data are largely in favor of functional constraint on TLRs between terrestrial ungulates and cetaceans indicating that the prevailing immune responses despite the difference in their respective habitats are a result of similar pathogenic pressure. However, we noted significant selection divergence in TLR3 suggestive of the possibility that dsRNA virus may have played a critical adaptive role in terrestrial ungulates and cetaceans divergence. In particular, divergent sites were evolving under accelerated rates in both clades but higher in cetacean clade (ω = 4.7) than in terrestrial ungulates (ω = 2.7) (Table 2). The result indicates potential adaptive response following water re-colonization and provide support for the growing appreciation of the significance of the RNA viruses in marine ecology [67, 68]. However, this result is somewhat paradoxical especially due to the fact that all RNA viruses known to infect cetaceans have thus far been single-stranded [67, 68]. Altogether, selection divergence in TLR3 and TLR7 (another viral-sensing RNA ranked second in terms of TLRs with the most number of positively selected sites), point to the increased significance of RNA viruses in the adaptations of terrestrial ungulates and cetaceans.

### TLR3 divergence and species-specific functional implications

Combining clade model analysis and giraffe substitution analysis on TLR3 structure allowed us to examine possible functional significance of terrestrial ungulates versus cetaceans TLR3 divergence with respect to particular species. TLR3 has previously been identified to show disparity in species-specific adaptive functional attributes between human and mouse [69]. This difference was associated with the narrow range of TLR3 functions in humans compared to the receptor broad range of functions in mouse. Our analysis indicated that such TLR3 species-specific functional attributes may also exist in some ungulate and cetacean species. TLR3-ECD contains fifteen N-linked glycosylation sites, all of which have been experimentally mutated individually or in pairs [56, 70]. Of particular interest was the unique giraffe N247D substitution occurring at the *N*-glycosylated site of TLR3-ECD. The certainty of this specific sequence change on giraffe TLR3 signaling mechanism will need validation experiments given that N247D mutation in human cell lines results in reduced or complete loss of activity. Although the N-glycosylation at this site does not seem to play any role in determining the conformational stability of the ECD crystal structure, it is likely that the linked glycan moiety may be involved in important cellular function related to TLR3, such as localization of the receptor to cellular compartments [70].

## Conclusions

The study has presented a molecular phylogenetic analysis of the seven TLR genes represented by giraffe, okapi, other terrestrial ungulates and cetaceans. The evidence of positive selection on the TLR genes reveals that pathogen mediated selective pressure may have shaped terrestrial ungulates and cetacean TLR evolution. The case for positive selection or selection divergence among or between terrestrial ungulates and cetaceans is supported by the correspondence of some of these sites to key TLR motifs including functionally relevant sites. The observed changes in TLRs are probably associated with different pathogenic environments that cetaceans and ungulates had to face during the course of their evolution. Sites under positive selection may have aided in their adaptation as they encountered novel environments. Further work, however, is required to ascertain the role of positively selected sites and other important substitutions identified in this study in relation to pathogen recognition. Furthermore, research is required to determine whether changes at positively selected sites and at other key sites translates to specificity in ligand recognition, signaling mechanism or differential susceptibility to pathogenic infections among ungulates and cetacean species.

## Additional files

Additional file 1: Giraffe and okapi TLRs coding gene sequences used in the analysis. A text file containing fasta formatted raw nucleotide sequences of giraffe and okapi that were used for all TLRs analyzed in the study. (TXT 37 kb)
Additional file 2: Selected species and accession numbers of all TLR sequences that were used in the analysis. An excel file containing the list of species and the associated RefSeq accession number of the sequence for each TLR. (XLS 13 kb)
Additional file 3: Table S1. Comparison of TLRs sequence characteristics (nucleotide, amino acids, Leucine-Rich-Repeats) between giraffe, okapi and cattle. Microsoft word document containing comparative sequence metrics between in all TLRs studied between giraffe, okapi and cattle. (DOCX 5 kb)
Additional file 4: Table S2 Mapping of significant positive selection sites that are concordant between M8 and SLR to key TLR domains and motifs. An excel file containing functionally important sites based on M8 and SLR predictions and showing correspondence of those sites with domain structure and LRR motifs of TLRs studied. (XLSX 10 kb)

## Abbreviations

AP-1: Activator protein 1
BEB: Bayes Empirical Bayes
CmC: Clade model C
CpG: Cytosine Phosphate Guanine
dsRNA: Double-stranded RNA
ECD: Extracellular domain
IRFs: Interferon regulatory factors
LPS: Lipopolysaccharides
LRT: Likelihood ratio test
LRR: Leucine Rich Repeats
MD2: Myeloid differentiation factor 2
MYD88: Myeloid differentiation primary-response protein 88
NF-kB: Nuclear Factor kappaB
PAML: Phylogenetic Analysis by Maximum Likelihood
PAMPs: Pathogen Associated Molecular Patterns
SMART: Simple Modular Architecture Research Tool
ssRNA: Single-stranded RNA
TIR: Toll/Interleukin-1 Receptor
TLRs: Toll-like receptors
TRIF: TIR domain-containing adaptor protein inducing IFNβ
