# Supplementary material for: Evolution of toll-like receptors in the context of terrestrial ungulates and cetaceans diversification
Source: BMC Evol Biol. 2017 Feb 16;17:54. doi: 10.1186/s12862-017-0901-7 (PMC5314619; doi:10.1186/s12862-017-0901-7)
Supplement: Additional file 3: Table S1. — Comparison of TLRs sequence characteristics (nucleotide, amino acids, Leucine-Rich-Repeats) between giraffe, okapi and cattle. Microsoft word document containing comparative sequence metrics between in all TLRs studied between giraffe, okapi and cattle. (DOCX 5 kb) [file 12862_2017_901_MOESM3_ESM.docx]

Table S1: Comparison of TLRs sequence characteristics (nucleotides, amino acids, Leucine-Rich Repeats) between giraffe, okapi and cattle

| **Name of TLR receptor (Giraffe protein length), Number of Giraffe Leucine Rich Repeats** | **Species** | **Amino acid % difference with giraffe sequence** | **Nucleotide % difference with giraffe sequence** | **Number of Okapi and Cattle Leucine Rich Repeats** |
| --- | --- | --- | --- | --- |
| TLR2 (784), 21 | Okapi | 1.5 | 1.1 | 19 |
|  | Cow | 5.2 | 3.7 | 21 |
| TLR3 (904), 21 | Okapi | 2.8 | 1.6 | 23 |
|  | Cow | 5.0 | 4.0 | 23 |
| TLR4 (841), 19 | Okapi | 1.4 | 1.4 | 19 |
|  | Cow | 5.7 | 4.0 | 21 |
| TLR5 (858), 21 | Okapi | 1.5 | 1.4 | 19 |
|  | Cow | 5.2 | 3.8 | 21 |
| TLR7 (1050), 25 | Okapi | 1.1 | 0.8 | 25 |
|  | Cow | 2.8 | 2.5 | 25 |
| TLR9 (1029), 25 | Okapi | 1.5 | 2.0 | 25 |
|  | Cow | 5.4 | 4.8 | 25 |
| TLR10 (812), 19 | Okapi | 2.6 | 1.7 | 19 |
|  | Cow | 4.8 | 3.5 | 19 |
